# Asymmetric ring structure of Vps4 required for ESCRT-III disassembly

**DOI:** 10.1038/ncomms9781

**Published:** 2015-12-03

**Authors:** Christophe Caillat, Pauline Macheboeuf, Yuanfei Wu, Andrew A. McCarthy, Elisabetta Boeri-Erba, Gregory Effantin, Heinrich G. Göttlinger, Winfried Weissenhorn, Patricia Renesto

**Affiliations:** 1 Unit of Virus-Host Cell interactions (UVHCI), University of Grenoble Alpes, F-38042 Grenoble, France; 2 CNRS, UVHCI, 71 avenue des Martyrs, F-38042 Grenoble, France; 3 Program in Gene Function and Expression, Program in Molecular Medicine, University of Massachusetts Medical School, Worcester, Massachusetts 01605, USA; 4 European Molecular Biology Laboratory (EMBL), Grenoble Outstation, 71 avenue des Martyrs, F-38042 Grenoble, France; 5 Institut de Biologie Structurale (IBS), University of Grenoble Alpes, 71 avenue des Martyrs, F-38044 Grenoble, France; 6 CNRS, IBS, 71 avenue des Martyrs, F-38044 Grenoble, France; 7 CEA, IBS, 71 avenue des Martyrs F-38044 Grenoble, France

## Abstract

The vacuolar protein sorting 4 AAA–ATPase (Vps4) recycles endosomal sorting complexes required for transport (ESCRT-III) polymers from cellular membranes. Here we present a 3.6-Å X-ray structure of ring-shaped Vps4 from *Metallosphera sedula* (MsVps4), seen as an asymmetric pseudohexamer. Conserved key interface residues are shown to be important for MsVps4 assembly, ATPase activity *in vitro*, ESCRT-III disassembly *in vitro* and HIV-1 budding. ADP binding leads to conformational changes within the protomer, which might propagate within the ring structure. All ATP-binding sites are accessible and the pseudohexamer binds six ATP with micromolar affinity *in vitro*. In contrast, ADP occupies one high-affinity and five low-affinity binding sites *in vitro*, consistent with conformational asymmetry induced on ATP hydrolysis. The structure represents a snapshot of an assembled Vps4 conformation and provides insight into the molecular motions the ring structure undergoes in a concerted action to couple ATP hydrolysis to ESCRT-III substrate disassembly.

## Abstract (web summary)

Vps4 is a AAA+ family protein involved in the disassembly of ESCRT-III polymers during membrane fission events such as occur during HIV budding. Here the authors propose a structure-based model of how the conformational flexibility of Vps4 can be translated into mechanical forces to disassemble ESCRT-III during membrane fission.

## Graphical abstract

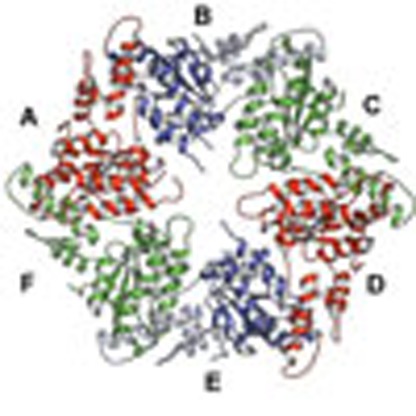

The endosomal sorting complex required for transport (ESCRT) machinery composed of ESCRT-0, -I, -II, -III and the vacuolar protein sorting 4 AAA–ATPase (Vps4) complex was originally discovered to catalyse plasma membrane receptor downregulation via multivesicular body formation^1^,^2^. Part of the ESCRT machinery including ESCRT-III and Vps4 are also recruited during cytokinesis^3^,^4^ and budding of some enveloped viruses to catalyse membrane fission^5^,^6^,^7^. ESCRT-III is thought to assemble into helical filaments within membrane necks of budding vesicles or viruses, or the cytokinetic midbody, to constrict it and set the stage for membrane fission^8^,^9^,^10^. Vps4, a member of the type-I or meiotic clade of AAA family of proteins (ATPase associated with various activities)^11^ disassembles ESCRT-III polymers from membranes^12^, which might be coupled to the membrane fission reaction itself^13^.

Proteins homologous to Vps4 and ESCRT-III are also present in the *Crenarchaeota* phylum of archaea where they catalyse final steps in cell division^14^,^15^, virus budding^16^ and vesicle release^17^.

Vps4 contains an amino-terminal microtubule interacting and trafficking (MIT) domain that interacts with carboxy-terminal peptide motifs termed MIT domain-interacting motif in activated eukaryotic and archaeal ESCRT-III proteins^4^. A linker region that varies in length between archaeal, yeast and mammalian Vps4 regulates its assembly and activity^18^, and connects to the large ATPase domain, followed by the small ATPase domain and a C-terminal helix that packs against the large subdomain^19^,^20^,^21^, thereby adopting a structure closely related to p97 (ref. 22) and spastin^23^. Nucleotide binds into a cleft between the large and the small ATPase domains and induces conformational changes^24^,^25^ associated with the ATPase reaction cycle^26^. Eukaryotic VPS4 has a β-domain inserted into the small ATPase domain^19^,^20^,^24^ that recruits the VPS4 activator Vta1p/LIP5 implicated in assembly and ATPase activity^21^,^27^,^28^,^29^,^30^. Crenarchaeal Vps4 lacks the β-domain^29^ and the regulatory factors are absent in *Crenarcheota*^31^. Although human cells have two paralogues of *vps4* (VPS4A and VPS4B)^32^, a single gene encoding Vps4 is present in archaea and yeast^2^.

Vps4 oligomerizes on nucleotide binding^12^ similar to other AAA-type ATPases^33^. Exceptions are crenarchaeal Vps4, which can form dodecamers in the absence of nucleotides at non-physiological protein concentrations^29^,^34^. Three cryo-electron microscopy structures of yeast Vps4 revealed two stacked ring structures with 12 or 14 subunits with substantial differences that might be attributed to different conformational states^35^,^36^,^37^. However, these structures were obtained with a catalytic inactive mutant (Glu233Gln) or in the presence of non-hydrolyzable ATP analogues. Recent work on archaeal and yeast Vps4 showed that wild-type proteins form hexamers in the presence of ATP, suggesting that the physiological oligomer is hexameric^29^. To date, however, only models of hexameric Vps4 exist that are based on the p97 hexamer structure^22^ and crystal packing contacts derived from sixfold screw axis crystal packing interactions^19^,^20^,^24^,^25^.

Here we characterized the oligomeric state of the Vps4 homologue from *M. sedula* (MsVps4) that belongs to the phylum *Crenarchaeota*. Although MsVps4 forms dodecamers at non-physiological protein concentrations in the absence of nucleotides, a MIT domain deletion mutant (MsVps4ΔMIT) assembles only into hexamers in solution. The catalytic activity of full-length MsVps4 is around ten times higher than that of MsVps4ΔMIT at physiological concentrations, consistent with a role for the MIT domain in ring assembly. The crystal structure of MsVps4ΔMIT revealed an asymmetric hexameric ring structure present in the asymmetric crystal unit. Further conformational changes occur on nucleotide binding as revealed by the structure in complex with ADP and Mg^2+^. The physiological relevance of two variable protomer interfaces was confirmed by site-directed mutagenesis *in vitro* and *in vivo* by probing oligomerization, ATPase activity, ESCRT-III disassembly and HIV-1 budding. We suggest that the structure may present a Vps4 intermediate state that is consistent with the conformational flexibility and asymmetry important for ATP-driven ESCRT-III disassembly.

## Results

### Oligomeric state of MsVps4 ΔMIT and enzymatic activity

**Figure 1:**
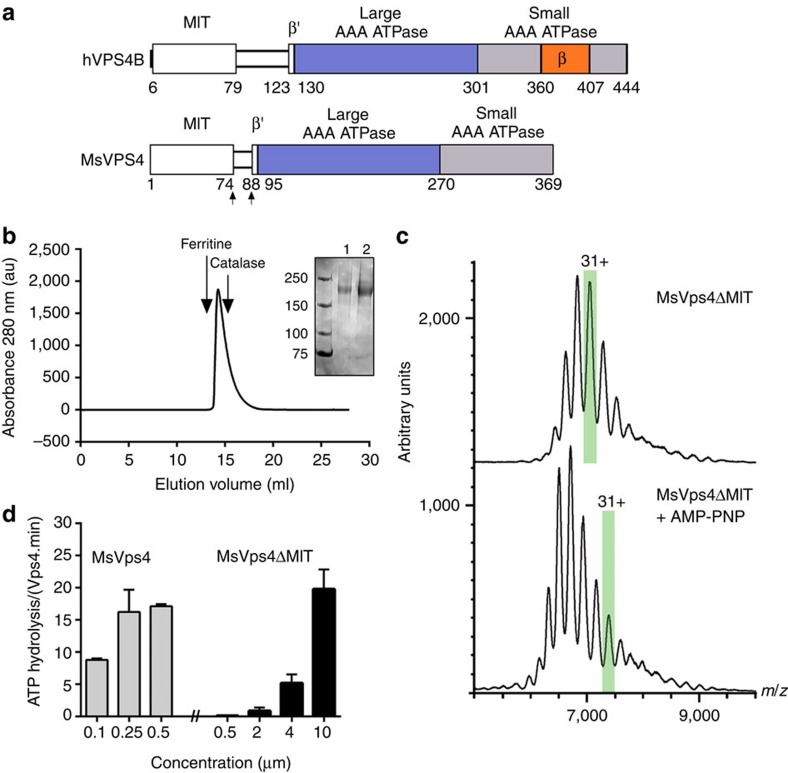
Biochemical and enzymatic analysis of recombinant MsVps4ΔMIT. (**a**) Domain organization of hVPS4B and MsVps4. MsVps4 was expressed as wild type or with N-terminal deletions (residues 75–369 and residues 88–369). (**b**) Elution profile of MsVps4ΔMIT (200 μM) from a Superose 6 SEC column. The elution peak of molecular sizing markers ferritin (450 kDa) and catalase (250 kDa) are indicated. Insert: SDS–PAGE analysis of MsVps4ΔMIT cross-linked with glutaraldehyde in the absence (lane 1) and the presence (lane 2) of 1 mM AMP-PNP and 1 mM Mg acetate. Hexamer formation was confirmed by native mass spectrometry. (**c**) Mass spectra of the cross-linked MsVps4ΔMIT in the absence (top spectrum) and the presence of AMP-PNP (bottom spectrum). MsVps4ΔMIT is hexameric in both conditions. The mass of MsVps4ΔMIT in the presence of AMP-PNP is larger than in the absence of the non-hydrolysable ATP analogue. This greater mass is most likely to be due to a covalent binding of the AMP-PNP. The ions carrying 31 charges (31+), highlighted in green, illustrate the mass difference between the two samples. (**d**) ATP hydrolysis of full-length MsVps4 and MsVps4ΔMIT at 60 °C. Values reported represent mean±s.d. of three independent experiments carried out on at least two different batches of purified Vps4.

Full-length MsVps4 as well as Vps4 from *Sulfolobus solfataricus* (SsoVps4) and *Acidianus hospitalis* (AhosVps4) form concentration-dependent dodecamers in the absence of nucleotides^29^,^34^; however, their oligomeric state is reduced to hexamers in the presence of ATP or ADP^29^. To determine the role of the N-terminal MIT domain in oligomerization, we purified a MIT domain deletion mutant that retained the linker region connecting the MIT domain to the ATPase domain (Fig. 1a and Supplementary Fig. 1). As for full-length protein, MsVps4ΔMIT (residues 75–369) oligomerizes in a concentration-dependent manner (Supplementary Fig. 2) without a 75 °C heating step applied during SsoVps4 and AhosVps4 purification^29^. The largest oligomers elute at 14.1±0.1 ml from a Superose 6 size-exclusion chromatography (SEC) column, at a position slightly bigger than the marker protein catalase (Fig. 1b). Chemical cross-linking of MsVps4ΔMIT in the absence and the presence of adenylyl imidodiphosphate (AMP-PNP) and Mg^2+^ revealed a band migrating at ∼200 kDa, indicating hexamer formation (Fig. 1b, right panel). This was confirmed by native mass spectrometry that produced a molecular weight (MW) of 201.56±0.01 kDa (calculated MW=201.36 kDa) and a slightly larger MW (211.77±0.01 kDa) in the presence of AMP-PNP and Mg^2+^(Fig. 1c). Although MsVps4ΔMIT hexamer formation can be observed at concentrations higher than 5–10 μM (Supplementary Fig. 2), no dodecamers are detected at concentrations up to 0.5 mM (20 mg ml^−1^), corroborating the importance of the MIT domain for dodecamer assembly *in vitro*.

Comparison of the ATPase activity of full-length MsVps4 versus MsVps4ΔMIT demonstrated that MsVps4ΔMIT is enzymatically active at 60 °C but requires a 40 times higher protein concentration. Although full-length MsVps4 was fully active at 0.25 μM with a turnover rate of ∼16 ATP per minute, MsVps4ΔMIT attained similar activity at 10 μM (Fig. 1d). From these data we conclude that the presence of the MIT domain most probably favours ATP-dependent oligomer formation and/or stabilization but is not essential for ATPase activity.

### Crystal structure of Vps4ΔMIT

**Figure 2:**
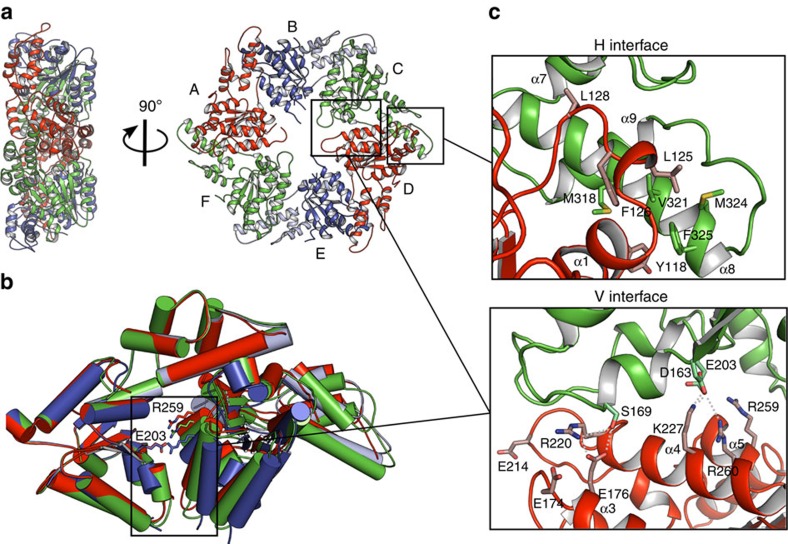
Crystal structure of the MsVps4ΔMIT pseudohexamer. (**a**) Cartoon representation of the MsVps4ΔMIT pseudohexamer viewed from two orientations. Asymmetric MsVps4ΔMIT has twofold non-crystallographic symmetry with opposing protomers making identical interactions. The protomers are labelled from A to F and identical protomers are represented in the same colour, except for protomers B and E, whose small ATPase domain is shown in light blue and the large ATPase domain in dark blue. (**b**) Superposition of the three different dimers present in the pseudohexamer, formed by A–B, B–C and C–D. The dimeric substructures are superimposed by aligning the large ATPase domain of the first protomer. This superposition reveals different positions of the large ATPase domain. The catalytic Glu203 (left subunit) and the arginine finger residue Arg259 (right subunit) are represented with sticks. (**c**) Molecular interactions at the H interface of the C–D dimer and molecular interactions at the variable V interface of the same dimer. A list of the molecular interactions of the three interfaces is shown in Supplementary Table 1.

The structure of MsVps4ΔMIT was solved by molecular replacement and single-wavelength anomalous diffraction (SAD) techniques, and the model was refined to a resolution of 3.6 Å (Table 1 and Supplementary Fig. 3a). The asymmetric unit of the P2_1_2_1_2_1_ space group contained one hexameric ring structure composed of the typical large ATPase domain and the small ATPase domain with its C-terminal helix packed against the large domain of the neighbouring protomer (Fig. 2a). The β-domain inserted into a loop of the small ATPase domain in eukaryotic Vps4 (Fig. 1a) is missing as in the related archaeal SsoVps4 homologue^29^. The 13 amino acid linker sequence plus 6 N-terminal residues that form a short β-strand (β′) in mammalian VPS4A and B are disordered in all protomers. The orientation of the large ATPase domain with respect to the small one is the same in all six protomers and their Cα atoms superimpose with a root mean squared deviation of 0.62 Å. The ring structure has twofold symmetry where opposing protomers make similar interactions (Fig. 2a). Three different protomer–protomer interfaces were identified between molecules A–B, B–C and C–D (interface D–E is the same as A–B; E–F corresponds to B–C and F–A to C–D), which are generated by a 10° and 15° rotation of the large ATPase domains in the different dimers (Fig. 2b). Each protomer–protomer interface can be split into two interacting surfaces, the conserved hydrophobic (H) interface between the small ATPase domain and the large ATPase domain from the neighbouring protomer (Fig. 2c), and the variable (V) interface between two large ATPase domains from neighbouring protomers (Fig. 2d). The H interface is hydrophobic and most of the interactions are conserved in all six interfaces (Supplementary Table 1). The V interface is dominated by polar interactions that vary substantially between the three different interprotomer interfaces (Supplementary Table 1). However, key interacting residues within the V and H interfaces are conserved between archaeal and human Vps4 (Supplementary Fig. 1). Superpositioning of the hexamer on itself by stepwise 60° rotations reveals the extensive conformational space that the pseudohexamer can adopt via its V and H interface in the absence of nucleotide (Supplementary Movie 1).

**Figure 3:**
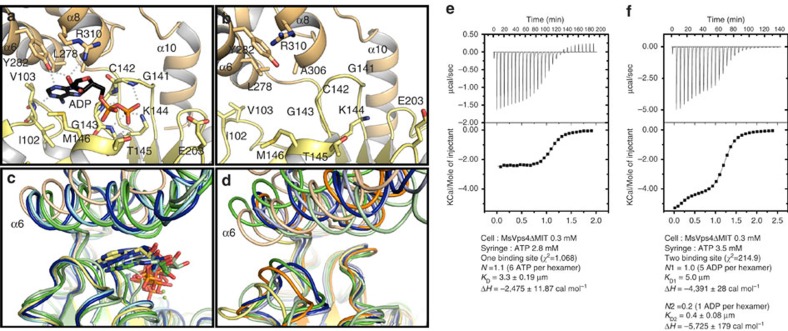
Crystal structure of MsVps4ΔL-MIT bound to ADP. View of the nucleotide-binding site. (**a**) Structure of ADP bound MsVps4ΔL-MIT. The MsVps4ΔL-MIT large ATPase domain (yellow) and small ATPase domain (beige) are represented in cartoon. ADP and interacting residues are shown as sticks and coloured by atom type. Polar interactions are indicated by grey dashes. (**b**) Close-up of the nucleotide-binding site of apo MsVps4ΔMIT (protomer F). (**c**) Superposition of the ADP-bound MsVps4ΔL-MIT (yellow and beige), ATPγS-bound yeast Vps4 (green, pdb 3EIH), ADP-bound yeast Vps4 (pale green, pdb 2QPA), ATP-bound mouse VPS4B (blue, pdb 2ZAN) and ADP-bound mouse VPS4B (pale blue, pdb 2ZAO). (**d**) Superposition of different Vps4 structures with no nucleotide bound. Superposition of apo MsVps4ΔMIT (yellow and beige), apo SsoVps4 (orange, pdb 4LGM, Cl^−^ bound in the P-loop), yeast Vps4p (green, pdb 3EIH, molecule C, ethylene glycol-Mg^2+^ bound in the P-loop), apo yeast Vps4p (pale green, pdb 2RKO), hVPS4B (blue, pdb 1XWI, SO_4_ bound in the P-loop) and mouse VPS4B (pale blue, pdb 2ZAM). The structures are superimposed by aligning the large ATPase domains. Nucleotides are represented in sticks in the same colour as the corresponding structures. (**e**) ITC analyses of ATP binding to MsVps4ΔMIT. ITC data were recorded on successive injection of ATP into the cell containing 300 μM MsVps4ΔMIT, which permits nucleotide-independent hexamer formation. This revealed a 1:1 binding mode for ATP. (**f**) ITC analyses of ADP binding to MsVps4ΔMIT at a concentration of 300 μM. The experimental data fit best to a two binding-site model with one high-affinity binding site and five lower-affinity binding sites. Experiments shown in **e** and **f** were repeated three times.

To probe the conformation on nucleotide binding, hexameric MsVps4ΔMIT and a shorter MsVps4 construct lacking the linker region (MsVps4ΔL-MIT, residues 88–369) were crystallized in the presence of ADP and Mg^2+^. Both constructs crystallized with P6_5_ helical symmetry (Table 1) with similar H interfaces as observed in the hexamer structure and a V interface close the to A–B interface. ADP is coordinated between the large and small ATPase domains (Fig. 3a and Supplementary Fig. 3b,c) and P**-loop residues Gly141, Cys142, Gly143, Lys144 and Thr145 (located on the large ATPase domain) bind the α- and β-phosphate groups of ADP, whereas Tyr282, Arg310 and Val103 (located on the small ATPase domain) make hydrogen bonds with the sugar and the base. The base is stacked between the small and large ATPase domain in a pocket composed of residues Met146, Leu278 and Ala306 (Fig. 3a). Comparison of the nucleotide-free apo structure present in the ring structure showed that ADP binding induced a 23° rotation of the small ATPase domain compared with the large one (Fig. 3b and Supplementary Movie 2). Aligning the large ATPase domain of MsVps4ΔMIT with those of yeast Vps4 bound to ATPγS and ADP, and mouse SKD1/VPS4B in complex with ATP and ADP^19^,^24^,^25^ showed significant differences in the position of the small ATPase domain including α6. ADP bound to MsVps4 occupies a similar position as ATPγS bound to yeast Vps4p, whereas ADP bound to yeast Vps4p penetrates less into the binding pocket (Fig. 3c). Substantial differences are also observed between all known Vps4 apo structures (Fig. 3d)^19^,^20^,^25^,^29^,^35^, representing the overall conformational flexibility of the nucleotide-free structure.

The nucleotide-binding cleft is open and accessible to nucleotides in all protomers of the apo structure. This is consistent with Isothermal titration calorimetry (ITC) experiments that demonstrated a 1:1 nucleotide binding ratio when ATP binding to MsVps4ΔMIT was determined at the MsVps4ΔMIT protein concentration that ensures nucleotide-independent hexamer formation *in vitro*. The overall *K*_D_ of ATP interaction is 3.3 μM (Fig. 3e). Measurement of ADP binding to MsVps4ΔMIT showed that the MsVps4ΔMIT hexamer binds five ADP with low affinity (*K*_D_=5.0 μM) and one ADP with higher affinity (*K*_D_=0.4 μM) (Fig. 3f). Together, these data indicate that MsVps4 can bind six ATP with low affinity and six ADP in a cooperative way *in vitro*.

### Key interface residues for Vps4 assembly and activity

**Figure 4:**
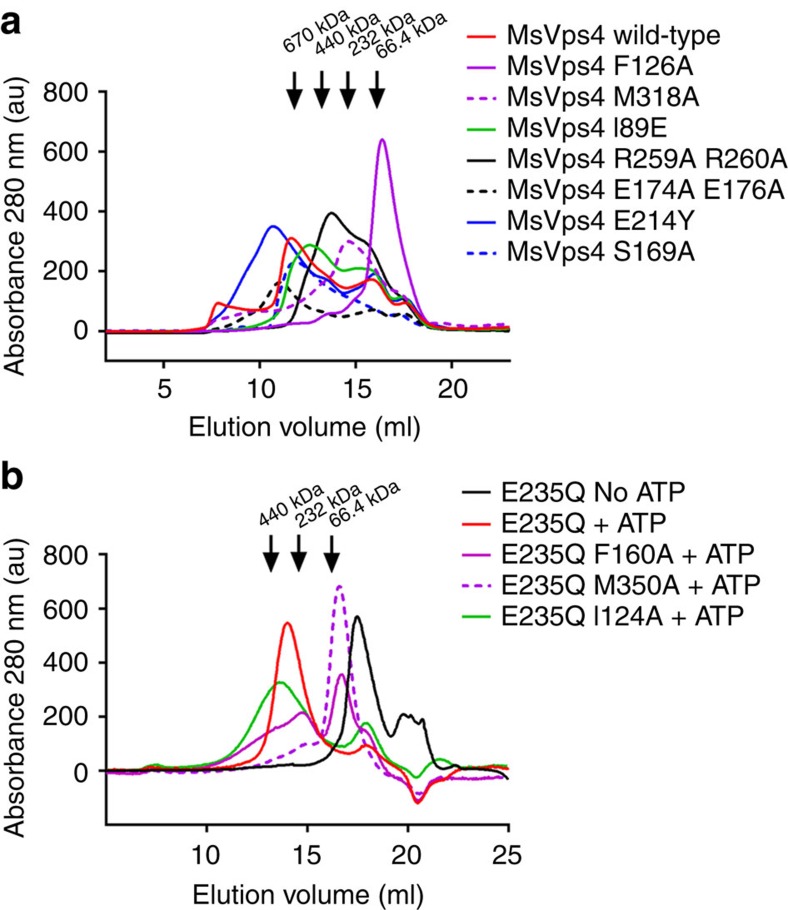
SEC analysis of MsVps4 and hVPS4B wild type and mutants. (**a**) Wild-type full-length MsVps4 and mutants thereof were separated on a Superose 6 SEC column at a protein concentration of 70 μM that induces dodecamer formation. The elution peak of molecular sizing markers thyroglobulin (660 kDa), ferritin (450 kDa), catalase (250 kDa) and BSA (66.4 kDa) are indicated (**b**) The catalytic mutant of hVPS4B (E235Q) and mutants thereof were separated on a Superose 6 column at a concentration of 100 μM and in the presence of 1 mM ATP and 5 mM MgCl_2_, which induces hexamer formation. The elution positions of marker proteins are indicated.

To validate the interactions present in the MsVps4ΔMIT hexamer, we mutated residues conserved between archaeal and eukaryotic Vps4 homologues. As removal of the His-tag from wild-type MsVps4 was rather inefficient, the experiments with the mutants were carried out with N-terminally His-tagged MsVps4. The oligomeric state of interface mutants was first tested by SEC. This revealed four main peaks corresponding to dodecamers, hexamers, dimers and monomers for wild-type MsVps4 (Fig. 4a). Notably, His-tagged MsVps4 has an approximately six times higher ATPase activity compared with untagged MsVps4, indicating that the presence of the tag seems to facilitate activation. First, we changed Phe126 and Met318 from the conserved H interface to alanine (Fig. 2c). SEC analyses of MsVps4 Phe126Ala showed a single peak corresponding to a monomer (Fig. 4a), which was confirmed by SEC–multi-angle laser light scattering (MALLS) (Supplementary Fig. 4). This indicates that the Phe side chain is crucial for oligomer formation and/or stability. Consistent with the defect in assembly, the Phe126Ala mutant has no ATPase activity (Table 2). In contrast, the Met318Ala mutation in close proximity to Phe126 (Fig. 2c) produces hexamers, dimers and monomers on SEC analysis, but dodecamer formation is impaired (Fig. 4a). Hexamer formation was confirmed by small angle X-ray scattering experiment (SAXS) analysis (Supplementary Fig. 5). MsVps4 Met318Ala revealed an Rg of 4.64 nM and a calculated *D*_max_ of 17.2 nM. As the Rg and *D*_max_ are smaller for the MsVps4ΔMIT structure (4.29 and 13.65 nm, respectively), we conclude that the MIT domain is flexibly linked and detached from the ring in this construct. Its ATPase activity is also enhanced by a factor of 1.5 compared with wild-type MsVps4 (Table 2), corroborating the proposal that dodecamer formation is not required for ATPase activity^29^.

Within the variable V interface, the conserved Glu176 hydrogen bonds to Ser169 only in the C–D dimer but not in the other dimer interfaces (A–B and D–E) (Fig. 2d and Supplementary Table 1). A double mutation of Glu176Ala and Glu174Ala within this variable V interface produced dodecamers, hexamers, dimers and monomers by SEC (Fig. 4a); however, its ATPase activity was almost completely abolished (Table 2). Vice versa, a Ser169Ala mutant formed dodecamers and hexamers as analysed by SEC (Fig. 4a) but still showed ATPase activity, suggesting that Glu174 is more important than Glu176 and its hydrogen bond to Ser169. Mutation of the two conserved Arg fingers, Arg259Ala and Arg260Ala, located within the V interface, abrogates dodecamer formation. However, the hexamers (and dimers and monomers) formed *in vitro* (Fig. 4a) have no ATPase activity (Table 2).

The N-terminal region comprising residues 88–95 is disordered in the ring structure but form a short β′-strand and α-helix in the ADP-bound monomer structure, thus possibly acting as a flexible arm helping to reorient the MIT domain and its bound substrate during the ATP cycle. Mutation of Ile89 to Glu located in the β′-strand produced hexamers, but no dodecamers by SEC, indicating a linker contribution in higher-order assembly (Fig. 4a), although the elution volume indicates a slightly larger hexameric structure, which is likely to be due to the displacement of the MIT domain, thereby increasing the hydrodynamic radius of the complex. However, its ATPase activity was only slightly reduced (Table 2). As substrate has been suggested to pass through the central pore of the ring structure, we mutated the pore loop 2 residue Glu214 to Tyr. SEC analysis revealed the same elution profile (dodedecamers, hexamers, dimers and monomers) as observed for wild-type MsVps4 (Fig. 4a); however, the ATPase activity was reduced to 8% of the wild-type activity (Table 2). In summary, our mutagenesis data confirm the role of conserved key residues for MsVps4 assembly and demonstrate that ring formation is not sufficient for ATPase activity, which is coupled to pore loop 2 and V interface residues.

As the above described key residues are conserved between archaeal and human VPS4 (A and B) (Supplementary Fig. 1 and Supplementary Table 1), we mutated VPS4B homologous positions of three key residues that affect MsVps4 assembly and its ATPase activity. To test the assembly of VPS4B *in vitro* in the presence of ATP, ATP hydrolysis must be inhibited. Therefore, the Phe160 (MsVps4 Phe126), Met350 (MsVps4 Met318) and Ile124 (MsVps4 Ile89) to alanine mutations were tested by SEC with a VPS4B Glu235Gln active site mutant. This showed that VPS4B Glu235Gln elutes as a monomer from a SEC column in the absence of ATP and as a hexamer in the presence of ATP (Fig. 4b). The Phe160 mutant is mostly monomeric, but still forms some higher-order oligomers (Fig. 4b), which are absent in case of MsVps4. Met350, which is juxtaposed to Phe160 of the neighbouring protomer in MsVps4 (Fig. 2c) and thus occupy the same hydrophobic pocket generated by hexamer assembly, does no longer oligomerize (Fig. 4b). Consistent with no significant oligomerization in the presence of ATP, both the Phe160 and Met350 mutant of wild-type VPS4B have no ATPase activity (Table 2). Together, the data on the Phe160 and Met350 mutants indicate that these hydrophobic interactions are essential for VPS4B assembly and thus ATPase activity. The mutant Ile124Glu (MsVps4 Ile89) within the N-terminal β'-strand still assembled into hexamers, which elute slightly larger from a SEC column than the wild-type (Fig.4b). However, in contrast to MsVps4 Ile89Glu, which still had ∼85% ATPase activity, its ATPase activity is reduced to ∼10% (Table 2).

### Residues important for HIV-1 budding and ESCRT-III disassembly

**Figure 5:**
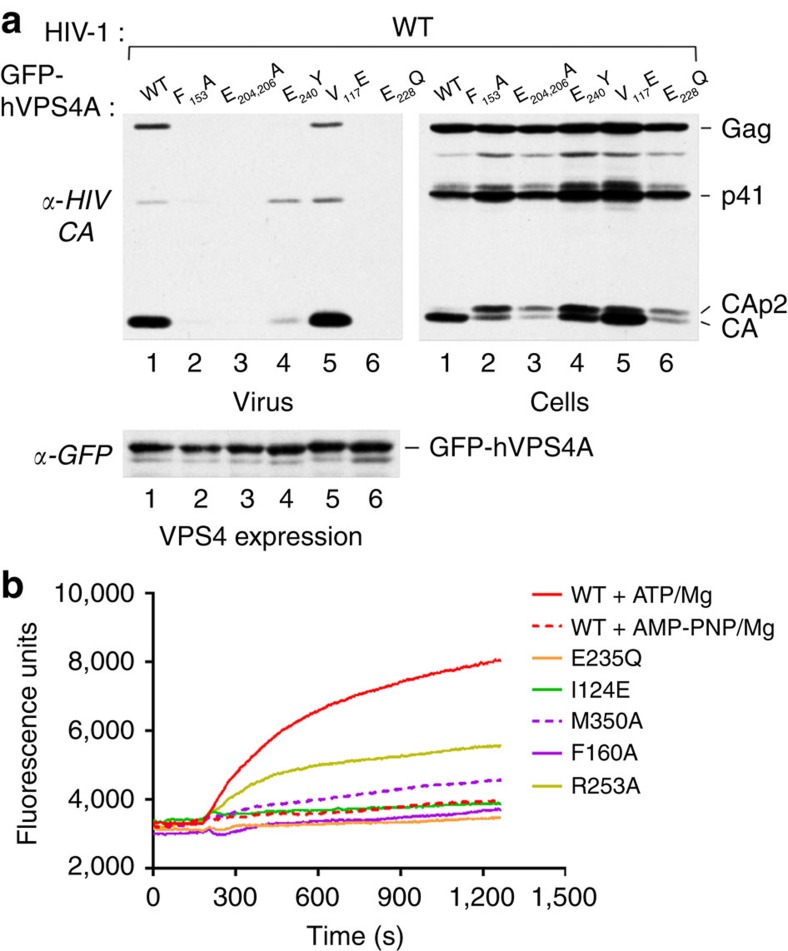
Mutations within hVps4 affect HIV-1 budding and ESCRT-III disassembly *in vitro*. (**a**) 293T cells were cotransfected with HIV-1 proviral DNA and vectors expressing GFP fused to wild-type (WT) hVps4A or to the indicated hVPS4A mutants. Virus release (upper left panel) and intracellular Gag processing were examined by western blotting with an anti- HIV-1 CA antibody (upper right panel). The expression levels of WT and mutant GFP–VPS4A were compared by western blotting with an anti-GFP antibody (lower panel). (**b**) Disassembly of fluorescein-labelled ESCRT-III CHMP2AΔC-CHMP3 helical tubes measured by change in fluorescence emission intensity on addition of VPS4B plus ATP and Mg^2+^ (red); MsVps4 plus AMP-PNP and Mg^2+^ (red dashed line) and mutants of hVPS4B plus ATP and Mg^2+^ as indicated (I124E, green; M350A magenta dashed line; F160, magenta; R253A, yellow). The experiment was repeated three times.

To further confirm the physiological relevance of the two interfaces, we generated hVPS4A mutants and compared their effect on HIV-1 budding with the dominant negative effect of catalytic inactive VPS4A (GFP–hVPS4A-Glu228Gln) as described previously^38^. Overexpression of the mutants that show no ATPase activity in the context of MsVps4 *in vitro*, Phe153Ala (MsVps4 Phe126), the double mutant Glu204Ala and Glu206Ala (MsVps4 Glu174 and 176), and the pore loop mutant Glu240Tyr (MsVps4 Glu214) in HIV-1-infected cells exerted a strong dominant negative effect on HIV-1 budding comparable to catalytic-inactive hVPS4A-Glu228Gln. No or very small amounts of Gag are detected in the cell culture supernatant and Gag processing is affected as evident from the presence of CAp2 and CA cleavage products detected in cells (Fig. 5a and Supplementary Fig. 6). In contrast, the Val117Glu mutation had no effect on virus budding despite the detection of CAp2 in cells (Fig. 5a and Supplementary Fig. 6). Expression levels of GFP–hVPS4A wild type and mutants were comparable in these experiments (Fig. 5a and Supplementary Fig. 6). We conclude that the key residues identified in affecting ATPase activity *in vitro* are also important for hVPS4A function in HIV-1 budding. In addition, the mutation of Val117 to Glu that disrupted dodecamer but not hexamer assembly had little effect on its ATPase activity *in vitro* (Table 2, MsVps4Ile89Glu) and no influence on HIV-1 budding, further indicating that dodecamer assembly is not essential for its physiological function.

As the dominant negative effect on HIV-1 budding observed with the above described mutants might be due to a defect in ESCRT-III disassembly, we tested a set of VPS4B mutants for their capacity to disassemble ESCRT-III CHMP2A-CHMP3 helical tubular structures *in vitro* as described before^39^. Fluorescein-labelled CHMP2A-CHMP3 tubes treated with wild-type VPS4B and AMP-PNP, and Mg^2+^ showed no changes in fluorescence emission intensity as a function of time (Fig. 5b). Dequenching, indicating disassembly of ESCRT-III CHMP2A-CHMP3, was measured as an increase in fluorescence emission when wild-type VPS4B was added together with ATP and Mg^2+^ (Fig. 5b). As expected, the catalytic mutant Glu235Gln did not disassemble the tubes, similar to the Ile124Glu and Phe160Ala mutants. The Met350Ala mutant had some disassembly activity and the Arg253Ala mutation within the V interface revealed an intermediate phenotype with ∼50% disassembly activity over 20 min (Fig. 5b). We conclude that VPS4-catalysed ESCRT-III disassembly requires conserved H and V interface residues.

## Discussion

Here we report the crystal structure of the hexameric AAA-type ATPase Vps4 from *M. sedula*. Although several structures of Vps4 have been reported^19^,^20^,^24^,^25^,^29^,^35^, none presented the native oligomeric conformation that is required for enzymatic activity^26^. The structure reveals an asymmetric pseudohexamer with three sets of opposing protomers making three different dimer–dimer interactions with neighbouring protomers. The same principles of asymmetry have been described for nucleotide-free and nucleotide-bound bacterial AAA-type ClpX ring structures with nucleotide-loaded and free protomers facing each other (Supplementary Fig. 7)^40^,^41^. Although the MsVps4 structure is nucleotide free, the conserved asymmetry indicates that opposing protomers may act together as reported for ClpX^40^,^41^.

AAA-type ATPases bind nucleotide at the interface between the large and small ATPase domain of one protomer and the large ATPase domain from an adjacent protomer, generating six binding sites^26^. ADP binding to MsVps4 induces rotation of the small ATPase domain with respect to the large domain as reported for other Vps4 structures^19^,^24^,^25^ and ClpX^40^,^41^. Although this rotation is facilitated by a single coil linking the large and small ATPase domains of ClpX, the two MsVps4 domains are connected by the corresponding linker and the C-terminal helix 10, which might render the monomer more rigid. Notably, comparison of MsVps4 and ClpX demonstrates that nucleotide binding induces a much larger rotation of the small ATPase domain of ClpX than of MsVps4 (Supplementary Fig. 7). ClpX hexamers have been reported to bind four nucleotides in solution^42^, although all six sites can also be occupied^41^. The crystal structure of MsVps4 shows that all nucleotide-binding sites are accessible and ITC analyses demonstrate that MsVps4 can bind six ATP molecules with low affinity at room temperature with no apparent cooperativity, similar to the p97-VCP D1 ring that also binds six ATP per hexamer^43^. ADP bound in a cooperative way to MsVps4 with at least one high-affinity and five low-affinity binding sites, at a MsVps4ΔMIT protein concentration that allows hexamer formation independent of nucleotide *in vitro*. Nevertheless, we cannot exclude the possibility that some hexamer assembly may have contributed to the ITC signal. MsVps4 nucleotide binding *in vitro* differs from data on ClpX, which was reported to have two high-affinity ATP-binding sites and two low-affinity sites at the nucleotide-loading step^41^. As the structures of ClpX adopt asymmetric conformations with four loaded and two unloaded states, it was suggested that the reaction cycle might pass via a four loaded, two unloaded state or alternatively via a five loaded, one unloaded state^40^,^41^,^44^.

Vps4 ring assembly requires nucleotides^20^,^29^, although archaeal Vps4 forms ring structures under non-physiological protein concentration and temperature conditions^29^,^34^. However, MsVps4 ring assembly is also induced by nucleotides under physiological conditions, because we measure significant ATPase activity at 0.25 μM concentration when MsVps4 is monomeric. As MsVps4 rings bind six nucleotides *in vitro*, we propose that binding of six nucleotides leads to assembly of the ring structure and might thus present the starting or an early intermediate conformation at the onset of the nucleotide reaction cycle. In case of eukaryotic VPS4, assembly is further regulated by Ist1 and Vta1 on ESCRT-III interaction, two factors that are absent in Crenarchaea^28^,^45^.

Both full-length MsVps4 and MsVps4ΔMIT exert ATPase activity *in vitro*, which is substantially higher in the presence of the MIT domain, in contrast to data reporting increased ATPase activity in linker and MIT domain deletion mutants^46^. However, the linker region of eukaryotic Vps4 was also reported to be important for ring assembly and activity^18^. Our crystal structures reveal that the β′-strand at the N terminus is ordered in the ADP-bound conformation and disordered in the nucleotide-free conformation, suggesting that this conformational flexibility might be important for MIT domain orientation. A mutation within the β′-strand abrogates MsVps4 dodecamer formation, but not hexamer formation and its ATPase activity is reduced. The corresponding VPS4B mutant also still forms hexamers, but its ATPase activity is greatly reduced, consistent with no ESCRT-III disassembly activity. The MIT domain is necessary to assemble MsVps4 dodecamers as its deletion produces only MsVps4 hexamers, which are enzymatically active, consistent with the proposal that the physiological unit of Vps4 is hexameric^29^. This is further validated by two MsVps4 mutants (Met318Ala and Ile89Glu) that are hexameric and have wild-type-like or slightly reduced ATPase activities. SAXS analysis of the MsVps4 Met318Ala mutant suggests hexameric ring structures with the MIT domains detached from the ring by the flexible linker. As the MIT domain auto-inhibits ESCRT-III engagement^47^, a conformation with the MIT domains assembled on top of the ring as observed by electron microscopy^35^,^36^,^37^ might constitute an intermediate state during the activation cycle. The question remains whether there is a physiological role for dodecameric Vps4 such as recruiting multiple Vps4 hexamers to the site of ESCRT-III function, thereby accelerating ESCRT-III disassembly. Notably, double ring structures of ClpA unfold proteins faster than single ring ClpX structures^48^.

The potential physiological relevance of the observed MsVps4 interfaces was confirmed by mutating V and H interface residues conserved between MsVps4, yeast Vps4 and human VPS4A and B. The effects of the mutations are twofold. They either influence VPS4 assembly leading to no or low ATPase activity or they have no affect on hexamer assembly, but are nevertheless physiologically inactive, because the mutations disturb the ATPase cycle, thereby inhibiting HIV-1 budding and ESCRT-III disassembly *in vitro*. The H interface mutant Phe126Ala (MsVps4) and its corresponding VPS4A and B mutants show defects in oligomerization, no ATPase activity, inhibit HIV-1 budding and ESCRT-III disassembly *in vitro*. The second H interface mutant Met318 (MsVps4), which form together with Phe126 a hydrophobic pocket, is still hexameric and exerts ATPase activity, whereas its human VPS4B (hVPS4B) counterpart is defective in hexamer assembly and thus exerts no ATPase activity. It shows some residual ESCRT-III disassembly activity *in vitro*, which is consistent with the proposal that ESCRT-III polymers stimulate VPS4 assembly^28^. Reduced ATPase activity and hexamer formation *in vitro* were also reported for H interface mutants of SsoVps4 (Tyr121Asp and Phe328Ala)^29^. Notably, the effect of mutations within the V interface is similar in MsVps4 and human VPS4. A double mutation in the V interface (Glu174Ala and Glu176Ala) impaired the ATPase activity and blocked HIV-1 budding. As the glutamate residues are ∼22 Å distant from the active site glutamate 203, their substitution by alanine might inhibit the propagation of signals from one protomer to the next during the ATPase cycle. The arginine fingers (Arg259 and 260) have been implicated in nucleotide binding and ATP hydrolysis^49^. The structure shows that Arg259 makes different polar contacts depending on the dimer, wheres Arg260 has no clear stabilizing role in the unliganded MsVps4 structure. Nevertheless, the double mutation prevents dodecamer, but not hexamer formation. However, the hexamers lack ATPase activity in agreement with a previous report on SsoVps4 (ref. 29). Pore loop 1 and 2 mutants have been implicated in exerting a dominant negative effect on HIV-1 budding^20^,^25^ and ESCRT-III disassembly^46^. Mutation of Glu214 (MsVps4) to Tyr had no effect on dodecamer and hexamer assembly, but reduced the ATPase activity to <10% and its corresponding mutation in VPS4A (Glu240Tyr) blocked HIV-1 budding almost completely, similar to the reported Glu240Ala mutation^25^. Recent work showed that this conserved glutamate residue affects substrate binding within the pore^47^. This is also consistent with the importance of pore loop residues for ESCRT-III unfolding when passing through the central pore^50^. The defect induced by the alanine and tyrosine mutations indicates that the negative charge of the glutamate is critical for the ATPase cycle and thus for ESCRT-III unfolding.

**Figure 6:**
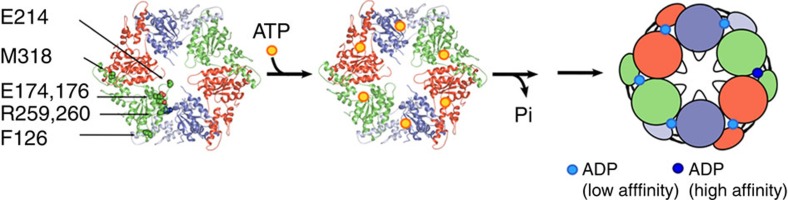
Model for Vps4 action. The MsVpsΔMIT pseudohexamer is represented as a cartoon, with identical protomers shown in the same colour. (left panel) The nucleotide-free pseudohexamer; (middle panel) the pseudohexamer may bind six ATP (yellow sphere), which are probably required to assemble the catalytic active ring structure; (right panel) ATP hydrolysis generates intermediate states, which leads at one point of the cycle to an intermediate conformation that binds one ADP with high affinity and five with low affinity. The position of the arginine fingers, the glutamate (174 and 176) V interface residues, the pore loop glutamate (214) and phenylalanine 126 are indicated. These residues are required for ATPase activity and most probably participate in the transfer of ATP hydrolysis-induced conformational changes of two opposing monomers to their neighbours, thereby powering the disassembly of ESCRT-III substrate complexes via the central pore^47^,^52^.

In summary, we identified several key residues that are located at the conserved V and H interfaces, far from the active site, but important for MsVps4 and human VPS4 function. Based on these observations, we propose the following mode of action for Vps4. First, Vps4 assembles in the presence of ATP molecules into a stable asymmetric ring structure that might resemble our nucleotide-free structure. ATP hydrolysis then leads to different intermediate states including potentially one when the ring has one high-affinity binding site and five low-affinity binding sites for ADP (Fig. 6). The different potential conformations adopted by the three opposing dimers coupled to the movement of the small ATPase domain on nucleotide binding and release indicates how these conformational motions might be propagated throughout the ring structure. Key interface residues such as Glu174 and Glu176 (located on α3), the arginine finger and the pore loop residue Glu214 are important for transferring the ATPase activity from two opposing protomers to their neighbouring set. This may generate flexing motions of the whole ring as suggested for ClpX^40^, whereby the nucleotide reaction cycle, free state, ATP binding, hydrolysis and ADP-bound states is converted into mechanical work^41^. The asymmetric structure of MsVps4 provides a snapshot of the conformational flexibility of a hexameric Vps4 AAA-type ATPase, which will help to understand its reaction cycle that is most probably similar to other asymmetric AAA-type ATPases functioning as proteases, helicases and unfoldases^51^,^52^,^53^.

## Methods

### Cloning of MsVps4 and hVPS4B

The gene encoding the full-length MsVps4 or truncated constructs were amplified by PCR from genomic DNA and using High-Fidelity PCR master mix (Phusion, Finnzymes) and gene-specific primers encoding a tobacco etch virus (TEV) site. *M*utations were introduced by overlapping PCR. The resulting products were cloned into the pDEST-17 vector using the Gateway cloning system from Invitrogen following the manufacturer's instructions. The gene encoding the full-length hVPS4B was cloned into the pProExHT vector. Mutations were introduced by overlapping PCR.The sequences of all constructs were confirmed by DNA sequencing.

### Purification of MsVps4 constructs

The *Escherichia coli* C41(DE3) strain was used as the host for protein expression. The cells were grown in lysogeny broth (LB) medium supplemented with ampicillin (100 μg ml^−1^) to an absorbance of ∼0.5 and expression was induced for 1 h at 37 °C by addition of 0.1 mM isopropyl-β-D-thiogalactopyranoside. To obtain selenomethionine (Se-Met)-incorporated MsVps4ΔMIT, cells were cultured in M9 medium supplemented with Se-Met (60 mg l^−1^) and other natural amino acids. Induction conditions were the same as for the native protein. The cells were pelleted by centrifugation (5,000*g*, 20 min), resuspended in lysis buffer (50 mM Tris pH 8.8, 50 mM NaCl, 10 mM imidazole, 1% CHAPS and Complete Protease Inhibitor (Roche Diagnostics)), then disrupted by sonication. After centrifugation (20,000 r.p.m., 30 min, 4 °C), the bacterial lysate was applied onto a Ni^2+^-NTA column (Qiagen) for affinity purification. After extensive washings (50 mM Tris pH 8.8, 50 mM NaCl and 20 mM imidazole), the protein was eluted in the presence of 300 mM imidazole. The fractions containing MsVps4ΔMIT were pooled and incubated overnight at room temperature with TEV (0.2 mg per 10 mg of protein) and dialysed against 50 mM Tris pH 8.8 and 50 mM NaCl. The TEV protease and uncleaved protein were removed by passing the solution through Ni^2+^-NTA column (Qiagen) and collecting the unbound protein. Cleaved MsVps4ΔMIT was then concentrated to 150–200 μM using an Amicon 400 concentrator with 30-kDa cutoff membrane and purified by anion-exchange chromatography (monoQ) and Superose 6 SEC on an ÄKTA FPLC system (GE Healthcare, UK). The peak fractions were analysed on 12% SDS–PAGE and Commassie Blue staining, and concentrated to 10 mg ml^−1^ for crystallization. Incorporation of Se-met was confirmed by mass spectrometry. Extensive crystallization trials that included 576 conditions in 96-well sitting-drop vapour diffusion format were performed on various constructs at the high-throughput crystallization facility of the EMBL Grenoble outstation. Following optimization, crystals used for diffraction studies were grown at 20 °C in hanging drop vapour diffusion plates. Wild-type and mutant MsVps4 were purified by the same protocol.

### Purification of hVPS4B constructs

The *E. coli* C41(DE3) strain was used as the host for protein expression. The cells were grown in lysogeny broth (LB) medium supplemented with ampicillin (100 μg ml^−1^) to an absorbance of ∼0.8 and expression was induced overnight at 20 °C by addition of 0.1 mM isopropyl-β-D-thiogalactopyranoside. The cells were pelleted by centrifugation (5,000*g*, 20 min), resuspended in lysis buffer (50 mM Tris pH 8.0, 150 mM NaCl, 10 mM imidazole and Complete Protease Inhibitor (Roche Diagnostics)), then disrupted by sonication. After centrifugation (20,000 r.p.m., 30 min, 4 °C), the bacterial lysate was applied onto a Ni^2+−^NTA column (Qiagen) for affinity purification. After extensive washing (50 mM Tris pH 8.0, 150 mM NaCl and 20 mM imidazole), the protein was eluted in presence of 300 mM imidazole. The fractions containing MsVps4ΔMIT were pooled and incubated overnight at room temperature with TEV (0.2 mg per 10 mg of protein) and dialysed against 25 mM HEPES, 150 mM NaCl and 10 mM imidazole. The TEV protease and uncleaved protein were removed by passing the solution through Ni^2+^-NTA column (Qiagen) and collecting the unbound protein. Cleaved MsVps4ΔMIT was concentrated to 80–150 μM using an Amicon 400 concentrator with 10-kDa cutoff membrane and purified by Superose 6 SEC on an ÄKTA FPLC system (GE Healthcare) in a buffer containing 25 mM HEPES pH 7.5, 150 mM NaCl and 0.5 mM dithiothreitol.

### Chemical cross-linking

Untagged MsVps4ΔMIT was purified by SEC (Superose 6) in 50 mM HEPES pH 7.5 and the fractions corresponding to oligomers were cross-linked for 20 min at room temperature with 0.05% glutaraldehyde, as reported for the yeast Vps4p complexes. Following addition of an equal volume of 1 M glycine pH 7 to quench the reaction, the sample was re-injected on the Superose 6 and peak fractions were collected for mass spectrometry.

### Mass spectrometry

Cross-linked samples were desalted and buffer exchanged using ultrafiltration (Vivaspin 500, Sartorius) with a 50-kDa cutoff, then diluted to a concentration of 5–8 μM in 250 mM NH_4_Ac at pH 7. Protein complex ions were generated using a nanoflow electrospray ionization source. Mass spectrometry analyses were carried out on a quadrupole time-of-flight mass spectrometer (Q-TOF Ultima, Waters Corporation, Manchester, UK). The instrument was modified for the detection of high masses. The following instrument parameters were used: capillary voltage up to 1.2–1.3 kV, cone potential=40 V, RF lens-1 potential=40 V, RF lens-2 potential=1 V, aperture-1 potential=0 V, collision energy=20–40 V and micro-channel plate=1,900 V. All mass spectra were calibrated externally using a solution of caesium iodide (6 mg ml^−1^ in 50% isopropanol) and were processed with the Masslynx 4.0 software (Waters Corporation).

### ATPase activity

The ATPase activity of the different constructs was carried out at 60 °C according to the manufacturer's instructions using the QuantichromeTM ATPase/GTPase Assay Kit (BioAssay Systems). Solutions containing 0.1–10 μM MsVps4, 25 mM HEPES pH 7.5, 50 mM NaCl, 2 mM MgCl2 and 1 mM ATP were prepared on ice. They were then incubated at 60 °C for various times (1–30 min). For hVPS4B, solutions containing 10 μM hVPSB, 25 mM HEPES pH 7.5 and 150 mM NaCl were prepared on ice; 10 mM MgCl_2_ and 1 mM ATP were added at the start of the experiment. They were then incubated at 37 °C for various times (10–30 min). The reaction was stopped on ice and reagent was added and incubated 30 min at room temperature. The complex formed by the reagent and free phosphate was detected by absorbance at 620 nm using a plate reader. A sodium phosphate standard curve was used to estimate the amount of phosphate released during ATP hydrolysis.

### Isothermal titration calorimetry

The binding constants of ATP to MsVps4ΔMIT were measured by ITC (MicroCal Inc.). All samples used in the ITC experiments were dialysed against 25 mM HEPES pH 7.5, 50 mM NaCl and 2 mM MgCl_2_. The ITC measurements were performed at 15°, 20° and 25 °C by making 28 injections (10 μl) of ATP or ADP to 1.4 ml of MsVps4ΔMIT. The concentrations of the MsVps4ΔMIT and ATP were 100 μM or 300 μM and 1 mM, respectively. Curve fitting was performed with the MicroCal Origin software. The experiments were carried out in triplicate.

### ESCRT-III CHMP2A-CHMP3 polymer disassembly

CHMP2AΔC/CHMP3 polymers were assembled as described previously^39^. For amine-specific labelling of the co-polymer, NHS ester (or succinimidyl ester) of Alexa Fluor 488 (Life Technologies) was dissolved in anhydrous dimethyl sulfoxide and added to the co-polymer solution in hepes buffered saline (HBS). The reaction was carried out at 4 °C overnight at a final concentration of 2 mg ml^−1^ of protein and 300 μM of the fluorophore. The reaction mixture was centrifuged and the pellet was extensively washed, to remove free fluorophore, and resuspended in HBS. The efficiency of the labelling was estimated by measuring the absorbance of the protein at 280 nm (taking in account the absorbance of the dye at 280 nm) and the fluorescence of Alexa Fluor 488 at 495 nm and the estimated value was ∼1.5 dye per CHMP2AΔC/CHMP3 dimer. The disassembly of the polymer was monitored on a BMG FLUOstar OPTIMA Microplate Reader. The different constructs of hVPS4B (10 μM) were mixed with the CHMP2AΔC/CHMP3 polymers (3 μM) in reaction volumes of 100 μl HBS. After ∼150 s, ATP/Mg (1 mM per 10 mM) was added to the mixtures containing hVPS4B wt, hVPS4B E235Q, hVPS4B I124E, hVPS4B M350A, hVPS4B F160A and hVPS4B R253A. After ∼150 s, AMPPNP/Mg (1 mM per 10 mM) was added to the mixture containing hVPS4B wt. Emission intensity was measured at 520 nm as a function of time by exciting the Alexa 488 at 490 nm.

### Mammalian expression plasmids and HIV-1 budding assay

The GFP–VPS4A mutations were generated via overlapping PCR and the correctness of the sequence was confirmed by DNA sequencing. To examine the effects of wild-type and mutated GFP–VPS4A on HIV-1 release, 293T (ATCC CRL-3216) cells (1.2 × 10^6^) were seeded into T25 flasks and transfected 24 h later using a calcium phosphate precipitation technique. The cultures were transfected with 1 μg Vpu-negative proviral DNA (HIV-1_HXB2_) together with expression vectors for wild-type or mutated GFP–VPS4A. The total amount of transfected DNA was brought to 8 μg with carrier DNA (pTZ18U). Twenty-four hours post transfection, the cells were lysed in radioimmunoprecipitation assay buffer (140 mM NaCl, 8 mM Na_2_HPO_4_, 2 mM NaH_2_PO_4_, 1% NP-40, 0.5% sodium deoxycholate and 0.05% SDS) and the culture supernatants were clarified by low-speed centrifugation and passed through 0.45-μm filters. Virions released into the medium were pelleted through 20% sucrose cushions by ultracentrifugation for 2 h at 27,000 r.p.m. and 4 °C in a Beckman SW41 rotor. Pelletable material and the cell lysates were analysed by SDS–PAGE and western blotting, using the anti-HIV CA antibody 183-H12-5C to detect Gag proteins. GFP–VPS4A was detected using an anti-green fluorescent antibody (GFP) antibody (Molecular Probes/Life Technologies).

### SAXS analysis of MsVps4

SAXS data collection and analysis. MsVPS4 M318A was purified as described above (His tag was cleaved) and used for the SAXS experiments. SAXS experiments were performed at the BM29 BioSAXS beamline at the ESRF (Grenoble, France). Data were collected using an online SEC. Five hundred microlitres of MsVPS4 M318A at 220 μM was injected on a Superose 6 10/300 GL, equilibrated in 50 mM Tris pH 8.8 and 50 mM NaCl. Data corresponding to the elution peek of MsVPS4 M318A were analysed; the data were normalized to the intensity of the incident beam, the scattering of the buffer was subtracted and the resulting intensities were scaled for concentration. Data processing and analysis were performed using the ATSAS package^54^ and MWs were estimated^55^. The final merged scattering data were further evaluated using PRIMUS^56^. The isotropic scattering intensity *I*(*q*) was transformed to the distance distribution function *P*(*r*) using the programme GNOM, which was also used to calculate the maximum dimensions of the particle, the *D*_max_ (ref. 57).

### SEC and MALLS

SEC was performed with a Superose 6. The column was equilibrated in 50 mM Tris pH 8.8 and 50 mM NaCl, and MsVps4 Phe126Ala was injected at 200 μM. The experiment was performed with MALLS using a DAWN-EOS detector (Wyatt Technology Corp., Santa Barbara, CA) and refractive index measurement using a RI2000 detector (Schambeck SFD). Light-scattering intensities were measured at different angles relative to the incident beam and analysis of the data was performed with the ASTRA software (Wyatt Technology Corp.).

### Crystallization and structure determination

MsVps4ΔMIT was crystallized in 20 mM Tris, 50 mM NaCl, 0.2 M sodium malonate pH 7 and19% polyethylene glycol 3,350 by mixing 1 μl of the reservoir to 1 μl of protein at 10.8 mg ml^−1^. The crystals were cryoprotected in the same conditions containing 25% ethylene glycol and flash cooled in liquid N_2_ before data collection. Se-Met derivatized crystals of MsVps4ΔMIT grew under the same conditions. A data set from a Se-Met crystal was collected at 0.97 Å wavelength to 3.6 Å resolution on the ID14-EH4 beam line at the European Synchrotron Radiation Facility (ESRF, Grenoble, France). Data were processed with the programme XDS^58^ and the crystals belong to space group P2_1_2_1_2_1_ (Table 1).

The structure was solved by molecular replacement using the programme Phaser^59^ and a Vps4 model generated with the Phenix Ensembler programme^60^ using human Vps4 (PDB code 1XWI), yeast Vps4 (PDB code 2RKO), yeast Vps4 (PDB code 2QP9) and yeast (PDB code 3EIH) as input models. The molecular replacement model was confirmed by the single anomalous dispersion method employing the data set collected at the peak wavelength (0.9791 Å). The phases were improved and initial model building was performed with the programme Crank2 (ref. 61) using both the anomalous signal and the molecular replacement solution described previously. Further model building was completed manually with Coot^62^ and the model was refined with REFMAC^63^ to an *R*_factor_ of 26.4% and *R*_free_ of 31.8%. The model contains chain A residues 97–369, chain B residues 98–367, chain C residues 96–367, chain D residues 97–369, chain E residues 96–367 and chain F residues 96–369. The N-terminal residues 75–95 were disordered in all protomers and 82.92/8.35% of the residues are within the most favoured and allowed regions of a Ramachandran plot^64^.

### Crystallization and structure solution of MsVps4 in complex with ADP

Two constructs of MsVps4 were crystallized with ADP: MsVps4ΔMIT (residues 75–369) and MsVps4ΔL-MIT (residues 88–369). Final ADP/MsVps4ΔL-MIT co-crystals were grown by mixing 1 μl of 8 mg ml^−1^ MsVps4ΔL-MIT, 5 mM ADP, 10 mM MgCl_2_ and 1 μl of the reservoir solution containing 40% (w/v) polyethylene glycol 200 and 100 mM Tris-HCl buffer (pH 8.8). Crystals were directly flash cooled in liquid N_2_ at 100 K. Data were collected on beamline BM14 at the ESRF at a wavelength of 0.9737 Å. Final ADP/MsVps4ΔMIT co-crystals were grown by mixing 1 μl of 10 mg ml^−1^ MsVps4ΔMIT, 5 mM ADP, 5 mM MgCl_2_ and 1 μl of the reservoir solution containing 1.4 M sodium/potassium phosphate pH 5.6 (1.26 M sodium dihydrogen phosphate monohydrate and 0.14 M dipotassium hydrogen phosphate) and 7.5% (v/v) glycerol. Crystals were soaked in the reservoir solution supplemented with 25% (v/v) glycerol and flash cooled in liquid N_2_ at 100 K. Data were collected on beam line ID23-2 at the ESRF.

Data were processed with the programme XDS^58^. Both crystals belong to space group P6_5_ but with different cell dimensions (Table 1). The structures were solved by molecular replacement using the programme Phaser^59^ and the apo MsVps4ΔMIT model. The model was rebuilt using COOT^62^ and refined using REFMAC^63^, Phenix^65^ and PDB_REDO web server^66^. Statistics for data reduction and structure refinement are presented in Table 1. The models were evaluated by using COOT and Phenix. In both structures the nucleotides gave clear electronic density and were present in all MsVps4 subunits. For the ADP/MsVps4ΔL-MIT complex, one molecule of ADP and one molecule of MsVps4ΔL-MIT containing residues 97–363 are present in the asymmetric unit. The structure was refined against 2.4 Å data to an *R*/*R*_free_ of 20.6/26.6% and 98.1/99.6% of the residues, respectively, and are within the most favoured and allowed regions of a Ramachandran plot. For the ADP/MsVps4ΔMIT complex, three molecules of ADP and three molecules of MsVps4ΔMIT are present in the asymmetric unit. MsVps4ΔL-MIT chain A contains residues 97–366, chain B contains residues 88–366 and chain C contains residues 90–366. The structure was refined against 3.2 Å data to an *R*/*R*_free_ of 22.9/26.8% and 96.9/99.9% of the residues, respectively, and are within the most favoured and allowed regions of a Ramachandran plot.

### Structure analysis

Molecular graphics figures were generated with PyMOL (W. Delano; http://www.pymol.org). Further details are described in the supplementary methods.

## Additional information

**Accession codes:** Co-ordinates and structure factors have been deposited in the Protein Data Bank with accession IDs 4D80, 4D81 and 4D82.

**How to cite this article:** Caillat, C. *et al*. Asymmetric ring structure of Vps4 required for ESCRT-III disassembly. *Nat. Commun.* 6:8781 doi: 10.1038/ncomms9781 (2015).

## Supplementary Material

Supplementary Information Supplementary Figures 1-7, Supplementary Table 1, Supplementary Methods and Supplementary References

Supplementary Movie 1 Conformational flexibility of the nucleotide-free pseudohexamer of MsVps4. The six-fold symmetry axis of the pseudohexamer was calculated with the program Galaxy/SymD 1. The pseudohexamer was then rotated onto itself successively by 60°, 120° and 180°. The movie was generated by morphing the overlaid conformations. (Kim, C., Basner, J. & Lee, B. Detecting internally symmetric protein structures. BMC bioinformatics 11, 303 (2010))

Supplementary Movie 2 Rotation of the small MsVps4 ATPase domain upon ADP binding. Morphing between one protomer of apo MsVps4?MIT and ADP bound MsVps4?L-MIT. The protein is represented in cartoon and some of the residues implicated in ADP binding (Tyr282, Arg310) and of the hydrophobic interface (Met146, leu278, Met318 and Phe325) are represented in grey sticks. ADP is represented in grey sticks.

## Figures and Tables

**Table 1:** Crystallographic data collection and refinement statistics.

	**MsVps4ΔMIT**	**ADP/MsVps4ΔL-MIT**	**ADP/MsVps4ΔMIT**
*Data collection*			
Space group	P212121	P65	P65
Cell dimensions			
*a, b, c* (Å)	99.70, 127.39, 191.23	65.27, 65.27, 112.5	151.19, 151.19, 98.59
α, β, γ (°)	90, 90, 90	90, 90, 120	90, 90, 120
Resolution (Å)	48–3.6 (3.8–3.6)^*^	2.4 (2.49–2.40)^*^	3.2 (3.4–3.2)^*^
*R*_merge_	9.1 (61.6)	5.8 (63.1)	14.4 (51)
*I/σI*	8.31 (1.76)	16.7 (2.3)	9.9 (3.1)
Completeness (%)	99.1 (99.6)	99.7 (99.7)	99.6 (99.8)
Redundancy	5.3 (5.5)	4.1 (4.2)	4.6 (4.7)
			
*Refinement*			
Resolution (Å)	3.6	2.4	3.2
No. reflections	27,368	10,642	21,217
*R*_work_*/R*_free_	26.4/31.8	21.2/26.4	22.9/26.8
No. atoms			
Protein	12,875	2,133	6,528
Ligand/ion	0	27	81
Water	0	7	12
*B*-factors			
Protein	142.61	55.8	53.5
Ligand/ion		43.1	43.1
Water		41.7	21.2
Root mean squared deviations			
Bond lengths (Å)	0.01	0.013	0.011
Bond angles (°)	1.716	1.59	1.54

One crystal was used for each structure. *Highest resolution shell is shown in parenthesis.

**Table 2:** Summary of oligomeric states, enzymatic activity, ESCRT-III disassembly and HIV-1 budding of MsVps4 and hVPS4A, and B constructs.

**MsVps4 and hVPS4 constructs**	**Domain**	**Elution volumes (ml)**				Complex*	**ATPase**†	**ESCRT-III**‡	**HIV-1**§
MsVps4_wild-type		11.6	13.4	15.8	17.1	Dodecamer	100%	—	—
hVPS4B_E235Q +ATP			14.1		17.8	Hexamer	—	—	—
hVPS4B_wild-type +ATP/Mg		—	—	—	—	—	100%	100%	
hVPS4A_wild type		—	—	—	—	—	—	—	100%
MsVps4_Phe126Ala	H				16.4	Monomer	0.0±0.1%	—	—
hVPS4B_E235Q_Phe160Ala+ATP	H		14.7	16.7	17.9	Monomer	—	—	—
hVPS4B_Phe160Ala+ATP/Mg	H	—	—	—	—	—	0.0±0.1%	Inhibition	—
hVPS4A_Phe153Ala	H	—	—	—	—	—	—	—	Inhibition
MsVps4_Met318Ala	H		13.1	14.6	17.2	Hexamer	154±2,5%	—	—
hVPS4B_E235Q_Met350Ala+ATP	H			14.3	16.6	Monomer		—	—
hVPS4B_Met350Ala+ATP/Mg	H	—	—	—	—	—	0.0±0.1%	∼20%	—
MsVps4_Ile89Glu	AAA		12.6	15.8	17.5	Dodecamer	85.6±16.4%	—	—
hVPS4B_E235Q_Ile124Glu+ATP	AAA		13.6		18	Hexamer	—	—	—
hVPS4B_Ile124Glu+ATP/Mg	AAA	—	—	—	—	—	8.10±1.95%	Inhibition	—
hVPS4A_Val117Glu	AAA	—	—	—	—	—	—	—	100%
MsVps4_Glu174Ala_Glu176Ala	V	10.5	13.7	16	17.6	Dodecamer	0.14±0.1%	—	—
hVPS4A_Glu204Ala_Glu206Ala	V	—	—	—	—	—	—	—	Inhibition
MsVps4_Arg259Ala_Arg260Ala	V		13.7	15.4	17.3	Hexamer	0.1±0.02%		—
MsVps4_Glu214Tyr	pore loop 2	10.7	13.5	15.9	17.6	Dodecamer	7.9±1.5%	—	—
hVPS4A_Glu240Tyr	pore loop 2	—	—	—	—	—	—	—	Inhibition
MsVps4_Ser169Ala	V	11.9	14.6	—	17.4	Dodecamer	14.5±3.2%	—	—
hVPS4B_Arg253Ala+ATP/Mg	V	—	—	—	—	—	69.5±5%	∼50%	—

^*^Main complex formed *in vitro*.

^†^ATPase activity; reported ATPase values correspond to the mean±s.d. of at least three distinct experiments carried out on two or more batches of MsVps4.

^‡^Disassembly of ESCRT-III CHMP2A-CHMP3 tubular structures.

^§^Inhibition of HIV-1 budding.
