# Supplementary material for: Asymmetric ring structure of Vps4 required for ESCRT-III disassembly
Source: Nat Commun. 2015 Dec 3;6:8781. doi: 10.1038/ncomms9781 (PMC4686814; doi:10.1038/ncomms9781)
Supplement: Supplementary Information — Supplementary Figures 1-7, Supplementary Table 1, Supplementary Methods and Supplementary References [file ncomms9781-s1.pdf]

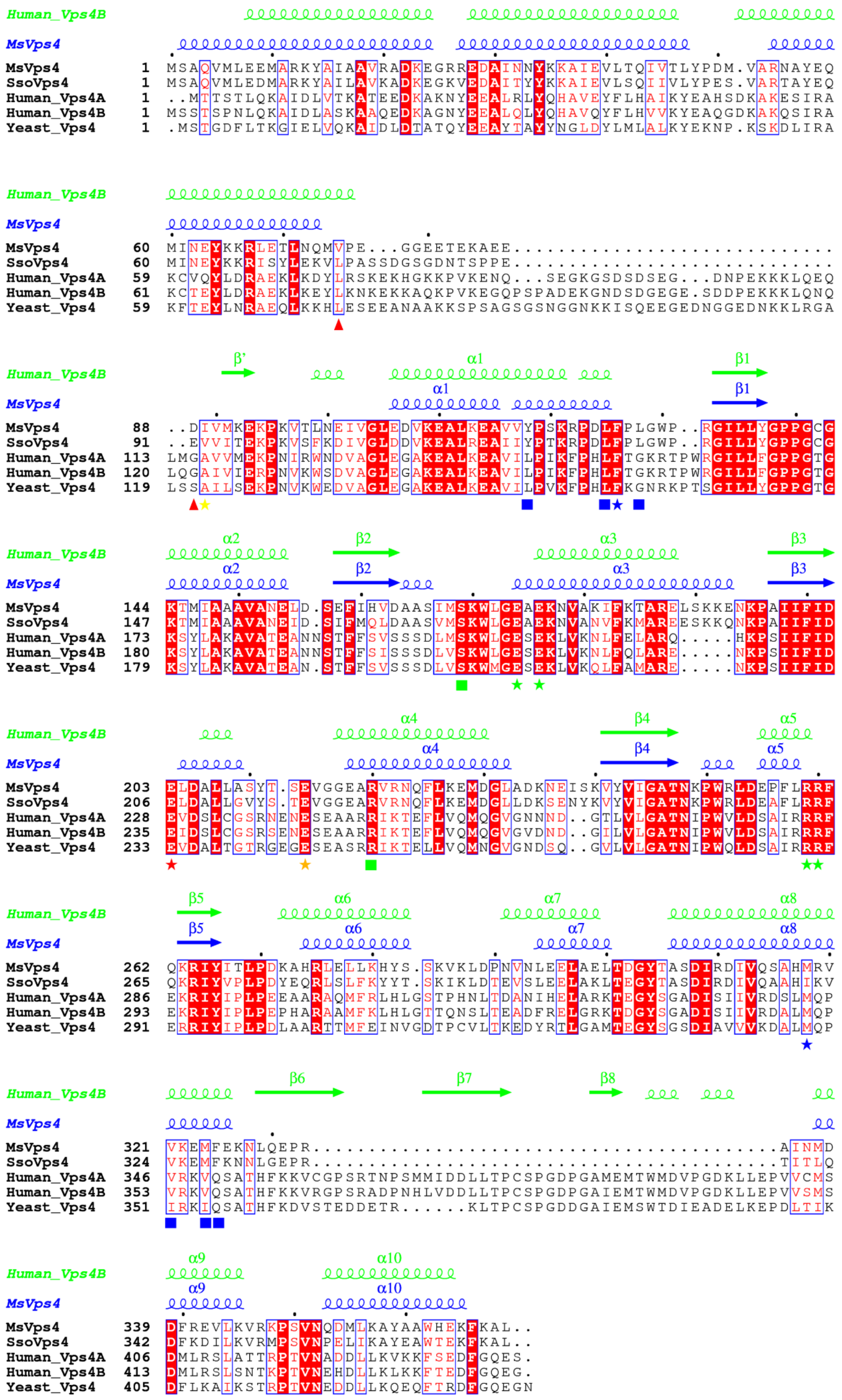

Human_Vps4B
MsVps4
MsVps4 1 MSAQVMLEEMARKYAIAAVRADKEGRREDAINNYKKAIEVLTQIVTLYPDM.VARNAYEQ
SsoVps4 1 MSAQVMLEDMARKYAILAVKADKEGKVEDAITYYKKAIEVLSQIIVLYPES.VARTAYEQ
Human_Vps4A 1 ..MTTSTLQKAIDLVTKATEEDKAKNYEEALRLYQHAVEYFLHAIKYEAHSDKAKESIRA
Human_Vps4B 1 MSSTSPNLQKAIDLASKAAQEDKAGNYEEALQLYQHAVQYFLHVVKYEAQGDKAKQSIRA
Yeast_Vps4 1 .MSTGDFLTKGIELVQKAIDLDTATQYEEAYTAYYNGLDYLMLALKYEKNP.KSKDLIRA
Human_Vps4B
MsVps4
MsVps4 60 MINEYKKRLETLNQMVPE...GGEETEKAEE............................
SsoVps4 60 MINEYKKRISYLEKVLPASSDGSGDNTSPPE............................
Human_Vps4A 59 KCVQYLDRAEKLKDYLRSKEKHGKKPVKENQ...SEGKGSDSDSEG...DNPEKKKLQEQ
Human_Vps4B 61 KCTEYLDRAEKLKEYLKNKEKKAQKPVKEGQPSPADEKGNDSDGEGE.SDDPEKKKLQNQ
Yeast_Vps4 59 KFTEYLNRAEQLKKHLESEEANAAKKSPSAGSGSNGGNKKISQEEGEDNGGEDNKKLRGA
β' α1 β1
Human_Vps4B
α1 β1
MsVps4
MsVps4 88 ..DIVMKEKPKVTLNEIVGLEDVKEALKEAVVYPSKRPDLFPLGWP..RGILLYGPPGCG
SsoVps4 91 ..EVVITEKPKVSFKDIVGLDDVKEALREAIIYPTKRPDLFPLGWP..RGILLYGPPGCG
Human_Vps4A 113 LMGAVVMEKPNIRWNDVAGLEGAKEALKEAVILPIKFPHLFTGKRTPWRGILLFGPPGTG
Human_Vps4B 120 LQGAIVIERPNVKWSDVAGLEGAKEALKEAVILPIKFPHLFTGKRTPWRGILLFGPPGTG
Yeast_Vps4 119 LSSAILSEKPNVKWEDVAGLEGAKEALKEAVILPVKFPHLFKGNRKPTSGILLYGPPGTG
α2 β2 α3 β3
Human_Vps4B
α2 β2 α3 β3
MsVps4
MsVps4 144 KTMIAAAVANELD.SEFIHVDAASIMSKWLGEAEKNVAKIFKTARELSKKENKPAIIFID
SsoVps4 147 KTMIAAAVANEID.SIFMQLDAASVMSKWLGEAEKNVANVFKMAREESKKQNKPAIIFID
Human_Vps4A 173 KSYLAKAVATEANNSTFFSVSSSDLMSKWLGESEKLVKNLFELARQ.....HKPSIIFID
Human_Vps4B 180 KSYLAKAVATEANNSTFFSISSSDLVSKWLGESEKLVKNLFQLARE.....NKPSIIFID
Yeast_Vps4 179 KSYLAKAVATEAN.STFFSVSSSDLVSKWMGESEKLVKQLFAMARE.....NKPSIIFID
α4 β4 α5
Human_Vps4B
α4 β4 α5
MsVps4
MsVps4 203 ELDALLASYT.SEVGGEARRVRNQFLKEMDGLADKNEISKVYVIGATNKPWRLDEPFLRRF
SsoVps4 206 ELDALLGVYS.TEVGGEARRVRNQFLKEMDGLLDKSENYKVYVIGATNKPWRLDEAFLRRF
Human_Vps4A 228 EVDSLCGSRNENESEAARRIKTEFLVQMQGVGNNND..GTLVLGATNIPWVLDSAIRRRF
Human_Vps4B 235 EIDSLCGSRSENESEAARRIKTEFLVQMQGVGVDND..GILVLGATNIPWVLDSAIRRRF
Yeast_Vps4 233 EVDALTGTRGEGESEASRRIKTELLVQMNGVGNDSQ..GVLVLGATNIPWQLDSAIRRRF
β5 α6 α7 α8
Human_Vps4B
β5 α6 α7 α8
MsVps4
MsVps4 262 QKRIYITLPDKAHRLELLKHYS.SKVKLDPNVNLEELAELTDGYTASDIRDIVQSAHMRV
SsoVps4 265 QKRIYVPLPDYEQRLSLFKYYT.SKIKLDTEVSLEELAKLTEGYTASDIRDIVQAAHIKV
Human_Vps4A 286 EKRIYIPLPEEAARAQMFRLHLGSTPHNLTDANIHELARKTEGYSGADISIIVRDSLMQP
Human_Vps4B 293 EKRIYIPLPEPHARAAMFKLHLGTTQNSLTEADFRELGRKTDGYSGADISIIVRDALMQP
Yeast_Vps4 291 ERRIYIPLPDLAARTTMFEINVGDTPCVLTKEDYRTLGAMTEGYSGSDIAVVVKDALMQP
β6 β7 β8
Human_Vps4B
MsVps4
MsVps4 321 VKEMFEKNLQEPR..AINMD
SsoVps4 324 VKEMFKNNLGEPR..TITLQ
Human_Vps4A 346 VRKVQSATHFKKVCGPSRTNPSMMIDDLLTPCSPGDPGAMEMTWMDVPGDKLLEPVVCMS
Human_Vps4B 353 VRKVQSATHFKKVRGPSRADPNHLVDDLLTPCSPGDPGAIEMTWMDVPGDKLLEPVVSMS
Yeast_Vps4 351 IRKIQSATHFKDVSTEDDETR......KLTPCSPGDDGAIEMSWTDIEADELKEPDLTIK
α9 α10
Human_Vps4B
α9 α10
MsVps4
MsVps4 339 DFREVLKVRKPSVNQDMLKAYAAWHEKFKAL..
SsoVps4 342 DFKDILKVRMPSVNPELIKAYEAWTEKFKAL..
Human_Vps4A 406 DMLRSLATTRPTVNADDLLKVKKFSEDFGQES.
Human_Vps4B 413 DMLRSLSNTKPTVNEHDLLKLKKFTEDFGQEG.
Yeast_Vps4 405 DFLKAIKSTRPTVNEDDLLKQEQFTRDFGQEGN

Supplementary Figure 1: Sequence alignment and secondary structure elements of MsVps4.

The sequences of MsVps4 (A4YHC5), SsoVps4 (Q97ZJ7), human VPS4A (Q9UN37), human VPS4B (O75351) and *S. cerevisiae* Vps4p (P52917) were aligned with ClustalX [1]. Residues Ile89, Phe126, Met318, E174, E176, Arg259, Arg260 and Glu214 are indicated by stars and red triangles indicate the N-terminal deletion constructs. Residues within the V interface are marked by green stars or squares and residues within the H interface are marked by blue stars or squares. The secondary structure elements of MsVps4 (blue) and human VPS4B (green, 1XWI) are shown.

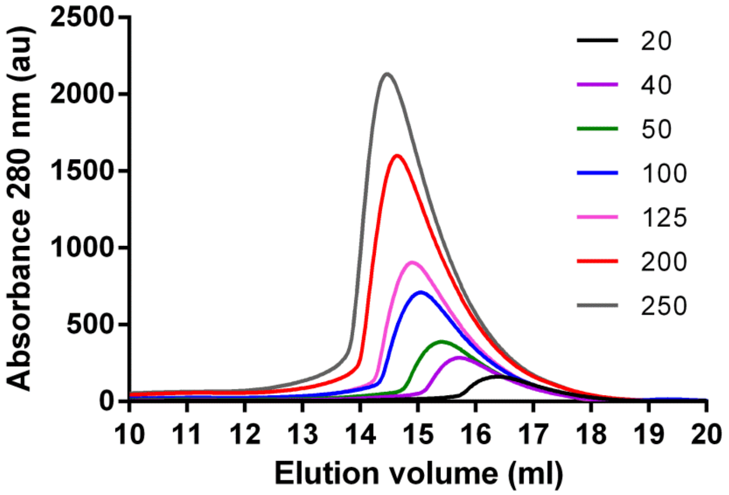

Supplementary Figure 2: Concentration-dependent oligomerization of MsVps4ΔMIT

The protein eluted from the Ni^{2+}-NTA column was digested by the TEV protease to remove the His-tag, then repurified over Ni^{2+}-NTA column and applied onto a Superose 6 column in a buffer containing 50 mM Tris 50 pH 8.8, 50 mM NaCl. 200 µl of MsVps4ΔMIT was injected at the indicated concentrations (20 to 250 µM). The minimal concentration still forming hexamers is 5 to 10 µM in the elution fraction.

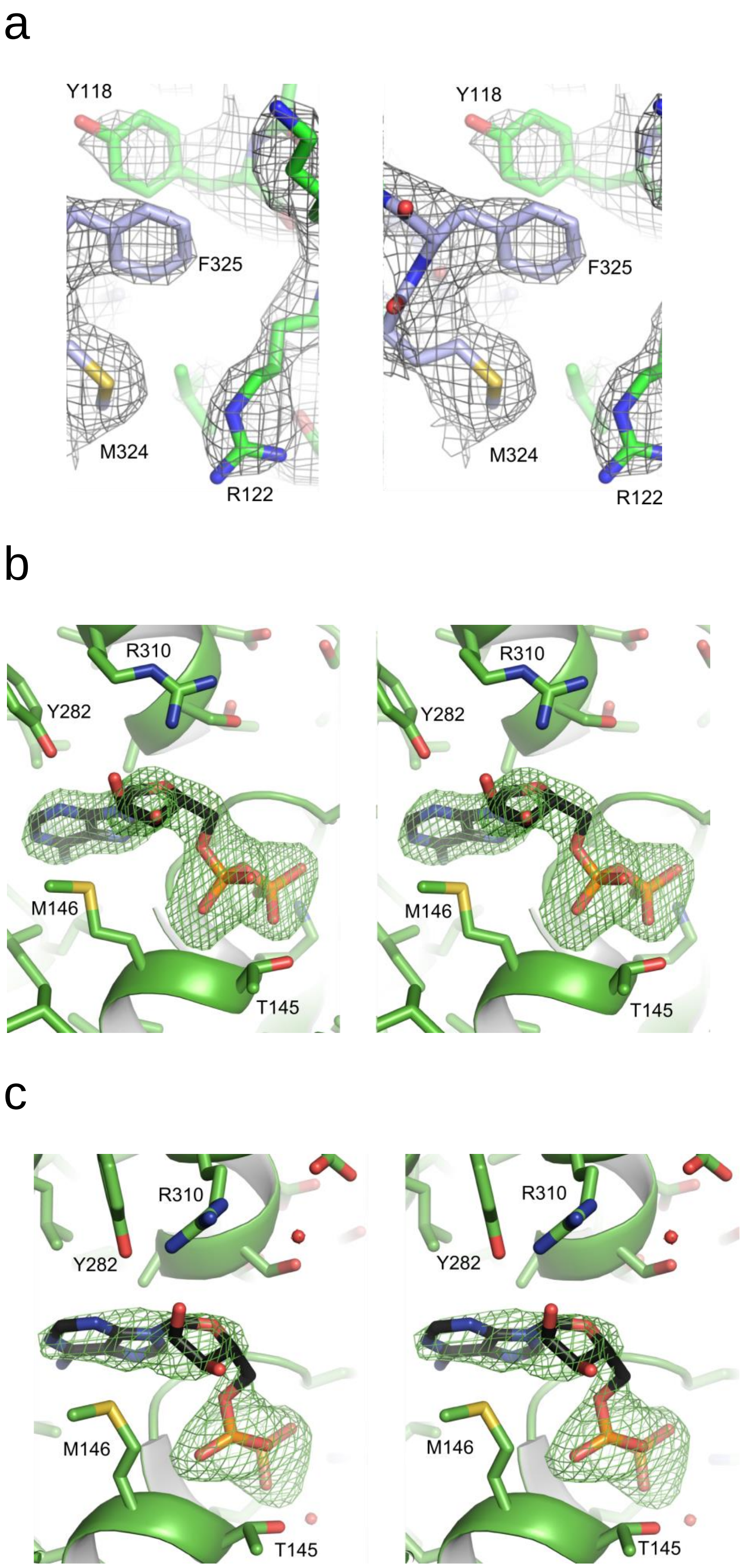

Supplementary Figure 3: Stereo images of electron density maps (a) Stereo image of the 2Fo-Fc electron density map (1.0σ) from the MsVPS4ΔMIT hexamer structure (4D80). (b)

Fo-Fc electron density omit map contoured at 3.0σ showing the density for ADP from the ADP/ MsVps4ΔL-MIT structure (4D81). (c) Fo-Fc electron density omit map contoured at 3.0σ revealing bound ADP in the ADP/ MsVps4ΔMIT structure (4D82).

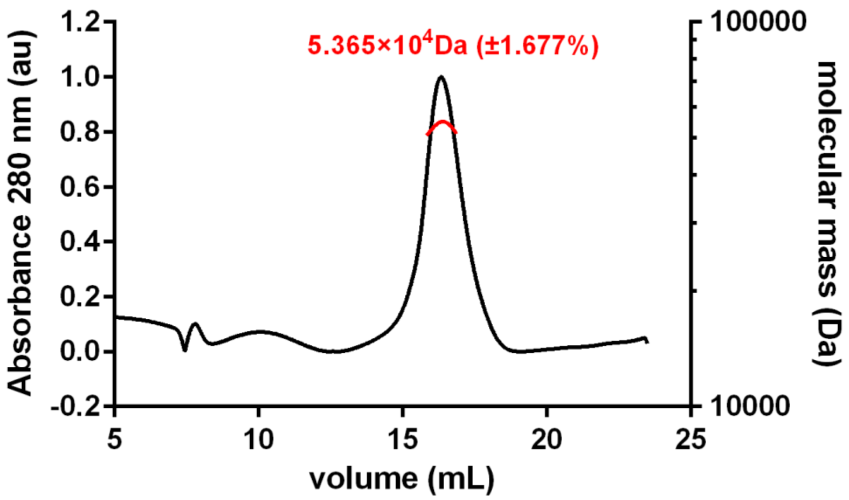

Supplementary Figure 4. SEC-MALLS analysis of MsVps4 Phe126Ala; SEC in combination with RI (refractive index) and MALLS (Multi Angle Laser Light Scattering) was performed with a superose 6 column. MsVps4 Phe126Ala (200 μM) elutes with the observed M_w of 53.65 ± 0.89 kDa, which is in agreement with a monomer conformation (calculated molecular mass = 45.46 kDa). The discrepancy between the observed and the calculated molecular weights might be explained by the fact that the MIT domain is linked via a flexible linker.

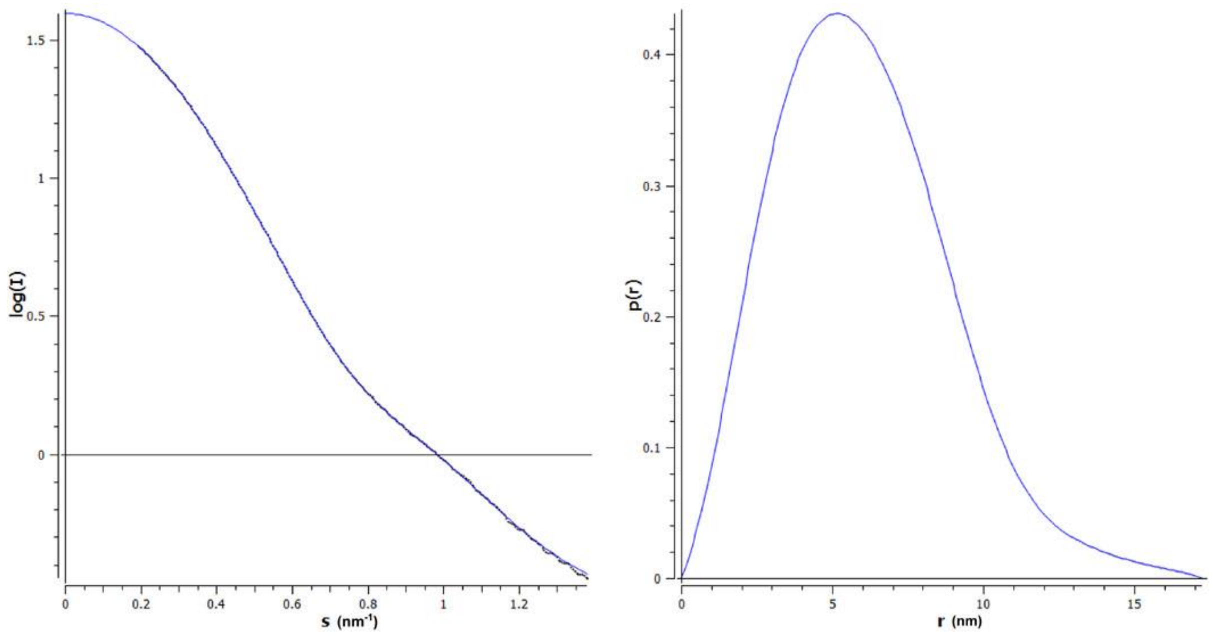

Supplementary Figure 5. SAXS analysis of MsVps4 Met318Ala. (left panel) Experimental scattering intensities (black line) obtained for MsVps4 Met318Ala are shown as a function of resolution and after averaging and subtraction of solvent scattering. The blue line represents the fit of the P(r) function to the experimental data. (right panel) The P(r) function indicates maximal dimensions (D_{max}) of 17.2 nm.

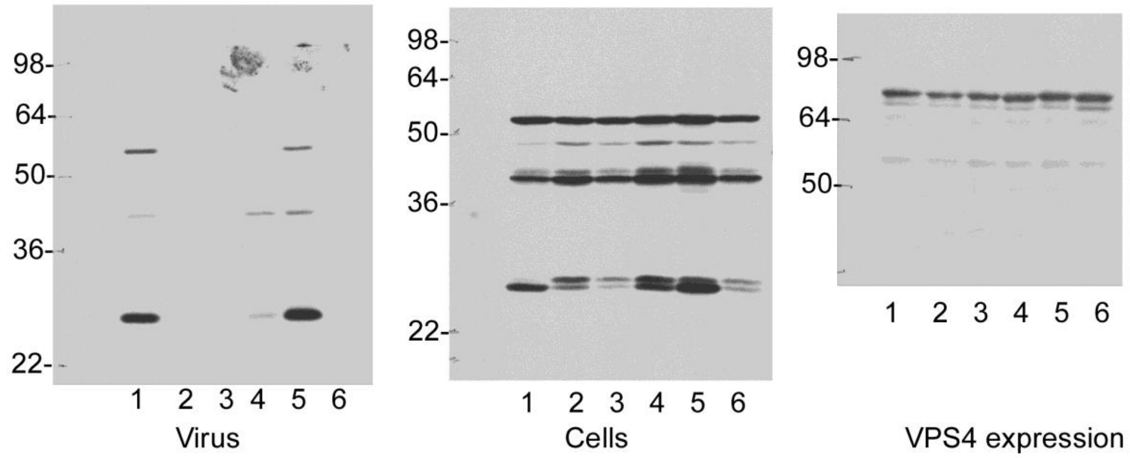

Supplementary Figure 6. Original western blots shown in Figure 5a. See text and Fig.5 for details. Lane 1, wild type GFP-VPS4A; lane 2, F153A; lane 3, E204A, E206A; lane 4, E240Y; lane 5, V117E; lane 6, E228Q.

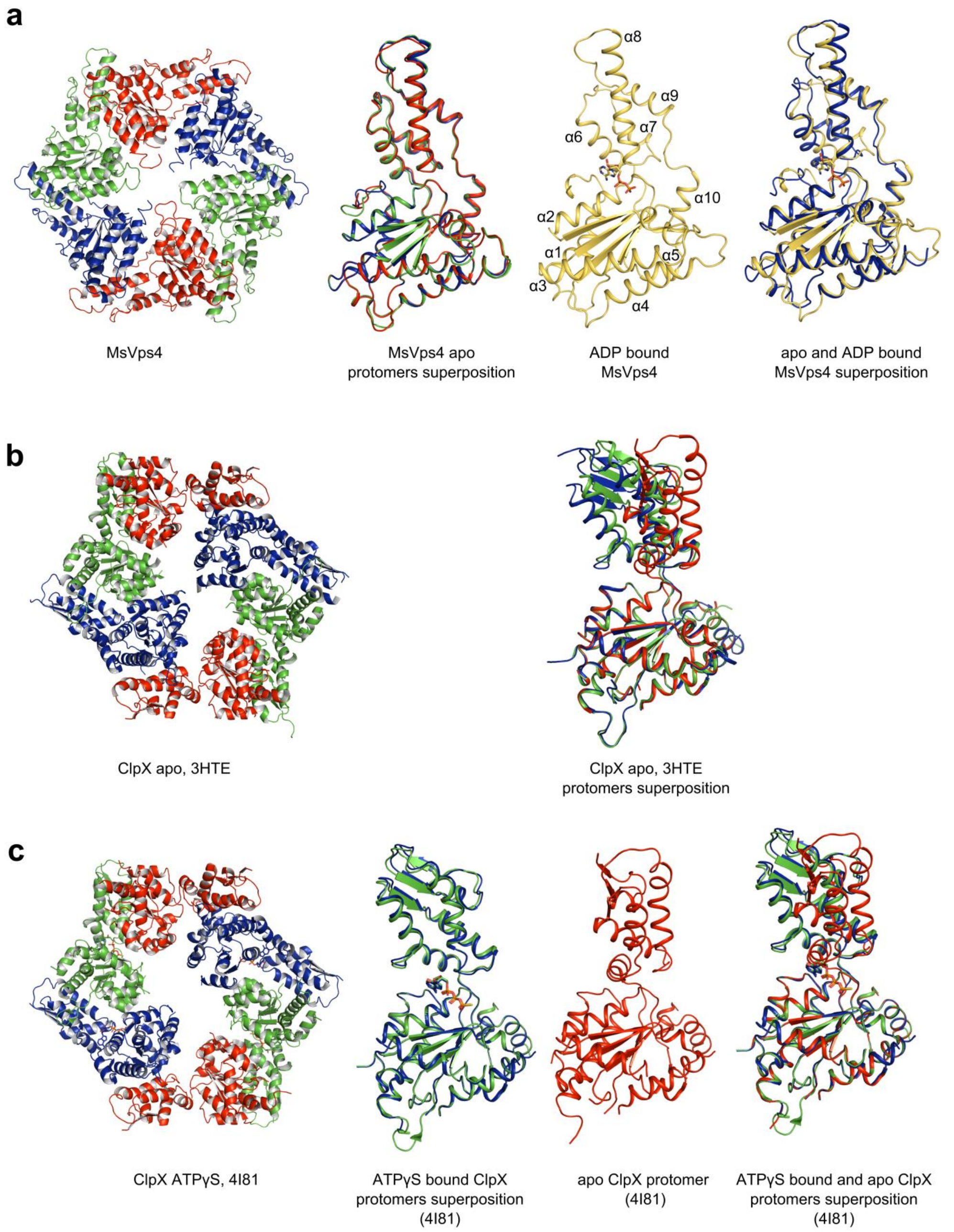

Supplementary Figure 7. Comparison of the pseudohexamers of ClpX and MsVps4.

a) MsVps4 nucleotide free pseudohexamer; Cα superpositioning of the three monomers form the dimers; MsVps4 bound to ADP and the Cα superpositioning of the nucleotide-free and the ADP-bound monomer showing the 24 º rotation of the small ATPase domain with respect to the large domain.

b) ClpX nucleotide-free pseudohexamer (pdb 3HTE); Cα superpositioning of the three monomers for the three dimers reveals that two are very similar (blue and green), while the third one (red) has its small ATPase domain rotated by 82º with respect to the large domain.

c) ClpX nucleotide-bound pseudohexamer (pdb 4I81); Cα superpositioning of the blue and green protomers in the ATPγS bound conformation have the same conformation; comparision with the nucleotide-free bound dimer (red) shows the same small ATPase domain rotation as in the complete nucleotide-free ClpX ring conformation (b).

		interface A-B		interface B-C		interface C-D	
		B-A	E-D	F-E	C-B	D-C	A-F
Interface Area (Å2)		947	1116	984	848	1259	1429
**Hydrophobic contact**							
Large Domain	Small Domain						
**Conserved interface (H)**		B-A	E-D	F-E	C-B	D-C	A-F
Tyr118		X	X	X	X	X	X
Leu125		X	X	X	X	X	X
Phe126		X	X	X	X	X	X
Leu128		X	X	X	X	X	X
	Tyr282	X	X				
	Val286			X	X	X	X
	His317			X	X	X	X
	Met318	X	X	X	X	X	X
	Val 321	X	X	X	X	X	X
	Met324	X	X	X	X	X	X
	Phe325	X	X	X	X	X	X
	Val346			X		X	X
	Met355	X	X			X	X
Phe366 (C-ter)							X
Leu369 (C-ter)				X		X	X
**Variable Interface (V)**							
	Leu172 (loop)		X			X	
	Val215 (loop)	X	X	X	X	X	X
**Hydrogen Bond**							
**Conserved interface (H)**		B-A	E-D	F-E	C-B	D-C	A-F
Gln314 NE2	Leu128 CO			3.34		3.07	3.12
Gln314 NE2	Gly129 CO			2.66	2.55	2.55	2.48
His317 NE2	Leu 125 CO			2.52	2.28	2.55	2.48
Lys322 NZ	Glu114 OE1 / OE2			3.44		2.91	3.48
Glu110 OE1	Glu 326 OE1				3.4		
Arg310 NH1	Leu128 CO			3.04	2.86		
Arg310 NH2	Leu128 CO			2.5	2.29		
Gln326 OE1	GLu110 OE1				2.24		
**Variable Interface (V)**		B-A	E-D	F-E	C-B	D-C	A-F
Lys227 NZ	Asp163 OD2					2.76	3.15
Lys227 NZ	Glu203 OE2						3.36
Glu176 OE2	Ser169 OG					3.52	3.31
Glu218 OE1	Ser213 OG		2.53				
Asn223 ND2	Ala165 CO			3.27			
Arg259 NH1/NH2	Glu203 OE2	2.6		2.38	2.41		
Val215 N	Glu214 OE2		3.46				
Arg220 NH1	Ser169 OG					3.86	3.37
**Salt bridge**							
**Conserved interface (H)**		B-A	E-D	F-E	C-B	D-C	A-F
Glu110 OE2	Lys322 NZ	3.77		3.89		2.91	3.63
Glu114 OE1	Lys322 NZ			3.44			2.48
Lys365 NZ	Asp354 OD2	3.86	3.75				
Lys365 NZ	Asp354 OD1		3.81				
**Variable Interface (V)**		B-A	E-D	F-E	C-B	D-C	A-F
Lys227 NZ	Asp163 OD1					3.75	3.92
Lys227 NZ	Asp163 OD2					2.76	3.15
Lys227 NZ	Glu203 OE2					3.88	3.36
Lys227 NZ	Glu203 OE1						3.68
Arg259 NE	Glu203 OE2	3.58	3.74				
Arg259 NH1	Glu203 OE2	2.6	3.63	2.38	2.41		
Arg259 NH1	Asp163 OD2	3.53	3.99				
Arg259 NH2	Asp163 OD2		3.53				
Arg260 NH2	Glu203 OE2			3.66	3.91	3.85	3.91

Supplementary Table 1

List of contact residues at the interface of two adjacent subunits. The residues are classified according to the type of interaction (hydrophobic interaction, hydrogen bond and salt bridge) and to their location in the conserved H interface (between the small ATPase domain and large ATPase domain) or in the V interface (between two adjacent large ATPase subunits), the residues in the left column correspond to the left subunit of the interface

(interface B for the B-A interface). For the hydrophobic contacts, only the residues with hydrophobic side chains buried in the interface are listed. For the salt bridges and hydrogen bonds the distances (in Å) between the corresponding atoms are indicated. The residues highlighted in green and in yellow are identical or similar to human VPS4A and B.

Supplementary Methods

Structure analysis. Domain motions in the different structures were analyzed using DynDom [2]. The C2 axis in the MsVps4ΔMIT hexamer was determined using Galaxy/SymD [3]. Subsequently the pseudohexamer was superimposed on itself in 60º steps and the morphing between the different superimposed conformations was performed with Chimera [4] and VMD [5]. RMSD calculations for superpositioned protomers were performed with the SuperPose server [6]. The sequence alignment was performed with the program ESPript [7].

Supplementary References

1. McWilliam, H. et al. Analysis Tool Web Services from the EMBL-EBI. *Nucleic Acids Res* **41**, W597-600 (2013).
2. Hayward, S., Kitao, A. & Berendsen, H.J. Model-free methods of analyzing domain motions in proteins from simulation: a comparison of normal mode analysis and molecular dynamics simulation of lysozyme. *Proteins* **27**, 425-37 (1997).
3. Kim, C., Basner, J. & Lee, B. Detecting internally symmetric protein structures. *BMC Bioinformatics* **11**, 303 (2010).
4. Pettersen, E.F. et al. UCSF Chimera--a visualization system for exploratory research and analysis. *J Comput Chem* **25**, 1605-12 (2004).
5. Humphrey, W., Dalke, A. & Schulten, K. VMD: visual molecular dynamics. *J Mol Graph* **14**, 33-8, 27-8 (1996).
6. Maiti, R., Van Domselaar, G.H., Zhang, H. & Wishart, D.S. SuperPose: a simple server for sophisticated structural superposition. *Nucleic Acids Res* **32**, W590-4 (2004).
7. Gouet, P., Courcelle, E., Stuart, D.I. & Metoz, F. ESPript: multiple sequence alignments in PostScript. *Bioinformatics* **15**, 305-8 (1999).
